# Droplet Impact on Superhydrophobic Surfaces Under High Pressures

**DOI:** 10.1002/smtd.202500913

**Published:** 2025-07-25

**Authors:** Yan Yan, Zhongqi Liu, Muhammad Amjad, Xiaolong Ma, Dongsheng Wen

**Affiliations:** ^1^ Institute of Thermodynamics Technical University of Munich 85748 Munich Germany

**Keywords:** droplet impact, droplet retention, gas entrapment, high pressure, superhydrophobic substrates

## Abstract

Though numerous studies on droplet impact have been conducted, the maximum ambient pressure reported is limited to 100 bar, and our understanding of droplet behavior under higher pressures remains unexplored. This study presents the first experimental investigation of droplet impact under high ambient pressure (up to 200 bar) onto different superhydrophobic substrates under low Weber number conditions. Four different regimes are identified, i.e., no bouncing, droplet bouncing with both satellite droplet retention and gas entrapment, droplet bouncing with gas entrapment, and complete droplet bouncing. The transition among different regimes is highly dependent on the ambient pressure and substrate topology. The droplet bouncing capability increases with the increase of ambient pressure, and complete bouncing is achieved for all substrates at P ≥ 175 bar. A phenomenological mode is developed taking into the consideration of both enhanced cushioning effect and hydrodynamic impact dynamics at high pressure. With a modified water hammer coefficient, the hydrodynamic impact model can be used to explain the disappearance of satellite droplet. Such work advances droplet study into 200 bar domain, which is of high relevance to a few high‐pressure applications such as deep sea oil/water separation.

## Graphical abstract

A phenomenological model of droplet impact under high ambient pressure (up to 200 bar) revealing unique phenomena of gas entrapment and satellite droplet retention, affected by substrate morphology and ambient pressure.

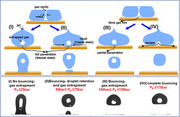

## 1 Introduction

Droplet impact on solid surface bears significant implications to a wide range of applications, including spray drying/cooling, diesel combustion, plasma spray coating, water proofing and self‐cleaning, anti‐icing and inkjet printing.^[^ ^1^, ^2^, ^3^, ^4^ ^]^ Quite a few applications are related to high pressures such as geothermal reservoir exploration, phase behavior of condensate in oil and gas reservoirs, high pressure combustion and spray, and subsea separation separations.^[^ ^5^, ^6^ ^]^ For instance, in subsea separation, efficient separation of multi‐phase hydrocarbons may lead to significant reduction in re‐compression requirements, providing savings in both capital and operational cost.^[^ ^5^ ^]^ For aerospace and automotive applications, fuel injection in the range of tens of MPa determines subsequent combustion and propulsion/power capability.^[^ ^6^ ^]^

Considering the importance of droplet impact, numerous experimental, theoretical, and numerical studies have been conducted, either under adiabatic or non‐adiabatic conditions. Some well‐known relationships have been established for droplet dynamics, such as the maximum spreading factor, the droplet contact time, and droplet rebound characteristics on different surfaces.^[^ ^1^, ^2^, ^7^ ^]^ One interesting phenomenon observed both experimentally and numerically is the droplet skating or bouncing from the substrate, which could happen on hydrophilic and smooth substrates,^[^ ^1^, ^8^ ^]^ on superhydrophobic substrates,^[^ ^2^, ^3^ ^]^ and on extremely hot substrates.^[^ ^9^ ^]^ The presence of gas, including both entrapped air and generated vapor via phase change, governs the droplet dynamics.^[^ ^10^, ^11^, ^12^ ^]^ It shall, however, be noted that most of these works have been conducted under atmospheric pressures, the effects of ambient pressure on droplet dynamics remain underexplored, especially under high pressures.

For sub‐atmospheric pressure studies, a pioneering study observed that under a few kPa range under adiabatic conditions, the typical droplet splash phenomenon can be largely suppressed due to the reduced or disappearance of entrapped gas film upon impact.^[^ ^13^, ^14^ ^]^ A few limited low‐pressure studies under heat transfer conditions are focused on stationary droplets.^[^ ^15^, ^16^, ^17^ ^]^ For instance, a droplet trampolining phenomenon was discovered as the pressure was reduced to a few kPa,^[^ ^15^ ^]^ where liquid droplets continuously bounced off from the substrate, like a trampolining. This was explained by a vapor overpressure effect, arising from the fast evaporation between the droplet and the substrate. A so‐called explosive bouncing, i.e., different to the Leidenfrost bouncing phenomenon, was reported on a heated silicon substrate as the ambient pressure was less than 6 kPa.^[^ ^17^ ^]^ However, due to the lack of proper control of the gas and substrate temperature in these studies, droplet experienced a non‐equilibrium phase change process during the rapid depressurisation, from possibly flash vaporization associated with the depressurization to a quasi‐equilibrium phase change due to temperature difference among the droplet, environment gas and the substrate. Such complexities make the interpretation of droplet dynamics difficult. We have recently investigated the droplet impact under low pressure conditions and discovered a unique pancake bouncing phenomenon with significantly reduced contact time.^[^ ^18^ ^]^ The experiments were performed in a low‐pressure environment chamber, and the pancake‐bouncing phenomenon was observed at environment pressure <2 kPa. As the droplet starts bouncing during the spreading stage, i.e., instead of in the receding period under 1 atm, the droplet contact time is significantly reduced in low pressure environment. The shortest contact time is identified as 1.72 ms in 0.5 kPa environment, which is an ∼86% reduction comparing to the 1 atm condition (12.2 ms). It should be noted that the pancake bouncing observed is fundamentally different from that reported in atmospheric conditions,^[^ ^19^ ^]^ even though they have similarities in droplet morphology and contact time reduction. All these studies suggest the importance of ambient pressure on droplet dynamics, particularly via the regulation of different gas dynamics.

Comparing to sub‐atmospheric studies, only very limited studies have been performed for droplet impact under high pressures.^[^ ^20^, ^21^ ^]^ A Lagrangian particle tracking was developed by Dupuy et. al.^[^ ^20^ ^]^ to investigate the impact of decane droplets toward dry and wet walls. The maximum pressure that was investigated was 97 bar due to the restriction of the experimental setup. Three flow regimes were observed, i.e., complete droplet deposition, partial deposition, and bouncing, under high pressures. It was also reported that high bouncing efficiencies was observed for droplet impacting onto wet substrates. However, due to the difficulties of visualization, no detailed impact phenomenon, such as complete spreading dynamics and gas entrapment phenomenon, could be identified. A few numerical simulations have also been conducted to investigate the impact and collision of droplets under an ambient pressure up to 150 bar, which revealed different scenarios comparing to that of under atmospheric conditions, such as the stochastic behavior of low inertia coalescence.^[^ ^21^ ^]^ It is expected that the increase of ambient pressure could have a lot of implications to the droplet dynamics by altering the properties of the surrounding gas and the interaction between the droplet and the substrate. The experimental study of droplet impact under extreme high pressures, i.e., beyond 100 bar, however, remains unexplored.

It is clear that our understanding of droplet impact behavior under high ambient pressures is highly limited. Equipped with a high‐pressure environmental chamber, we present in this work the first experimental investigation of droplet impact at pressures up to 200 bar under low Weber number conditions. A number of substrates with different wettability are designed and 3D printed. The influences of ambient pressure and substrate structure on droplet impact dynamics onto different superhydrophobic substrates are experimentally investigated. Four different regimes are identified for droplet impact on superhydrophobic substrates, i.e., no bouncing, droplet bouncing with both satellite droplet retention and gas entrapment, droplet bouncing with gas entrapment, and complete droplet bouncing. The disappearance of satellite droplet retention and gas entrapment inside droplet are highly dependent on ambient pressure and substrate structures, particularly the spacing distance of microstructures of the substrates. Such work advances the understanding of high‐pressure droplet dynamics and provides physical insights for surface design for high‐pressure applications.

## 2 Results and Discussions

### 2.1 General Observation: Ambient Pressure Effect

**Figure 1:**
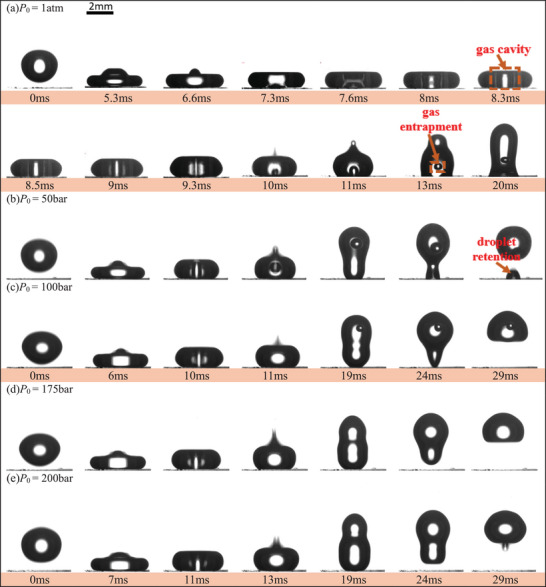
Time‐lapse images of droplet impact on micropillar substrate with *p* = 70 µm in different ambient pressures (1 atm, 50 bar, 100 bar, 175 bar, and 200 bar), showing the disappearance of satellite droplets and gas entrapments with the increase of ambient pressure. (a) *P*_0_ = 1 atm, We = 7.1, no bouncing with gas entrapment in droplet. (b) *P*_0_ = 50 bar, We = 6.5, bouncing with both satellite droplet retention and gas entrapment. (c) *P*_0_ = 100 bar, We = 6.1, bouncing with gas entrapment. (d, e) *P*_0_ = 175 bar and 200 bar, We = 5.7 and 5.5, completely bouncing. (The impact height is fixed and the slight variation of Weber number is due to the change of thermophysical properties, as detailed in the Supporting information).

**Figure** **1** (Video S1) displays the typical results of a substrate with [*a*, *p*, *h*] = [20,70,80] µm with a solid fraction of *f* = 0.082 under a range of ambient pressures, *P*_0_ = 1atm, 50, 100, 175, and 200 bar. The static, advancing and receding contact angles are θ_s_ = 166.4 ±  1.17°, θ_a_ = 170.4 ±  3.61° and θ_r_ = 158.5±2.81°, respectively.

Under *P*_0_ = 1atm, even the substrate has a high liquid repellence capablity, the whole droplet cannot be bounced, and is sticky on the substrate with a gas bubble entrapped inside, as shown in Figure 1a. Such results are consistent with a few other observations that the formation of entrapped gas inside an impacting droplet occurred at certain We number range under atmospheric pressure.^[^ ^22^ ^]^ However, as the ambient pressure increases, the droplet bouncing capability increases. Droplet can rebound when the ambient pressure ≥50 bar, as shown in Figure 1b–e. At *P*_0_ = 50 bar, satellite droplet retention and gas bubble entrapment phenomena can be observed, as shown in Figure 1b. As the pressure increases further, both phenomena become weakened. At *P*_0_ = 100 bar, the satellite droplet retention phenomenon is disappeared, Figure 1c, and at *P*_0_ = 175 and 200 bar, complete bouncing is observed, with no satellite droplet retention and no gas entrapment, Figure 1d,e.

**Figure 2:**
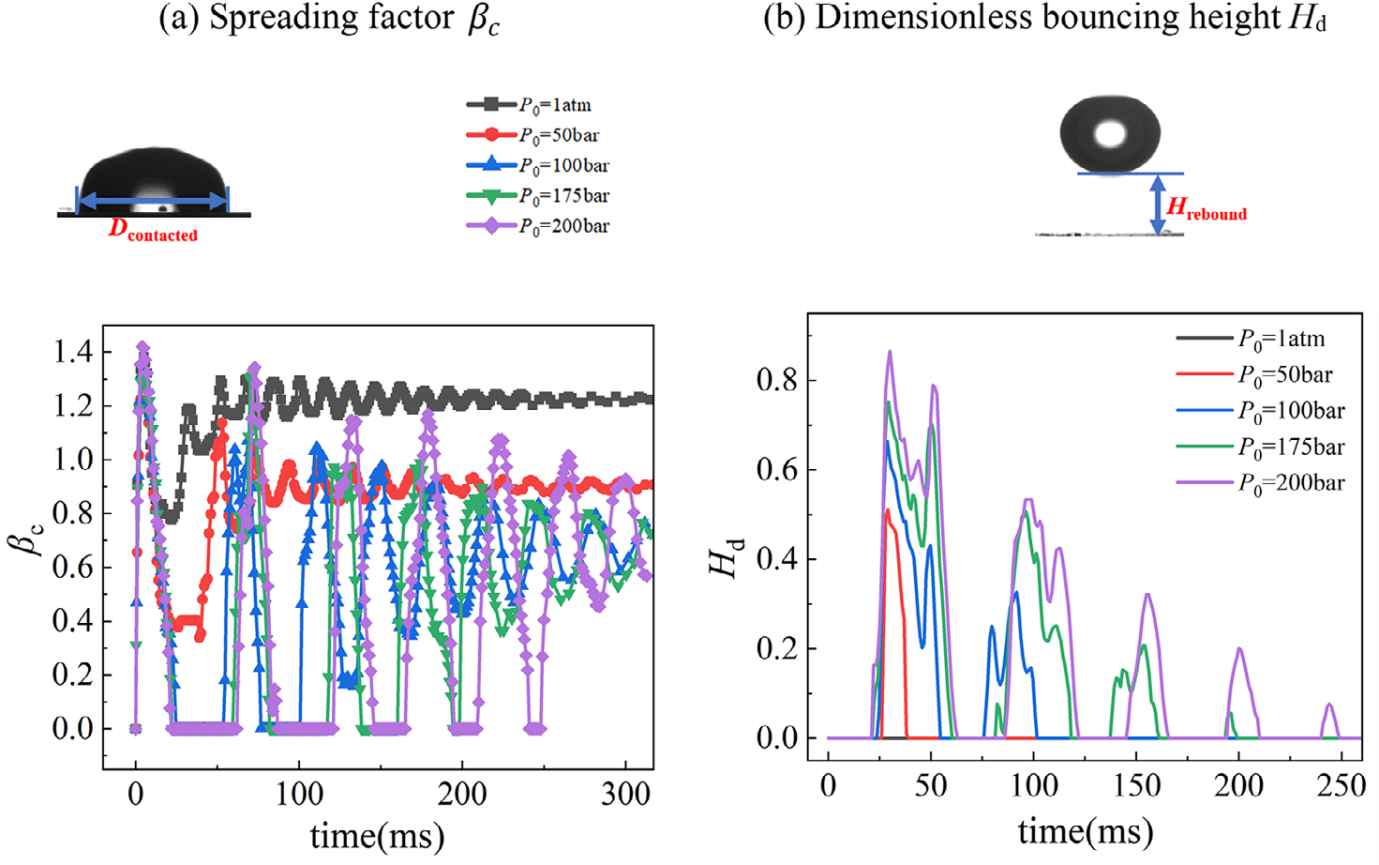
Quantitative descriptions (a) spreading factor β_*c*_and b) dimensionless bouncing height *H*_d_) of droplet dynamics on the micropillar substrate with *p* = 70 µm in different ambient pressure *P*_0_ (1atm, 50 bar, 100 bar, 175 bar, and 200 bar).

The quantitative descriptions of droplet dynamics in terms of spreading factor β_*c*_= *D*_contacted_/*D*_0_, and dimensionless bouncing height, *H*_d_ = *H*_rebound_/*D*_0_, are illustrated in **Figure** **2**. The presentation is based on statistical average of three events. Comparing to what happens under 1atm, where droplet fluctuates with a decaying amplitude after impacting on the substrate, large fluctuations are observed at high ambient pressures, Figure 2a. The decreases of β_*c*_ at *P*_0_ = 50 bar indicates droplet bouncing with a large satellite droplet retention, and the value drops to zero shows the bouncing of droplet without any droplet retention, as shown by *P*_0_ ≥ 100 bar. It also observed that except the case of 200 bar, β_*c*_shows slightly smaller values comparing to that under 1 atm conditions, probably due to air drag effect associated with the increased air density and statistical uncertainty. Figure 2b shows that droplet bounces higher and more frequently with increasing ambient pressure, where 1, 2, 4 and 5 times bouncing are observed at 50, 100, 175 and 200 bar, respectively. Clearly increased ambient pressure contributes greatly to the repellency of droplet from the substrate.

### 2.2 Effect of Substrate Topology

**Figure 3:**
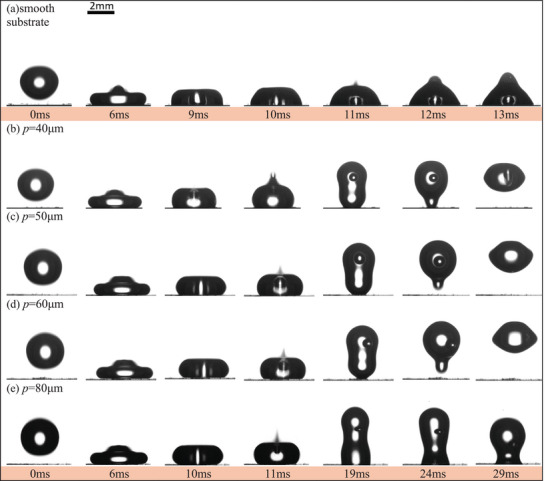
Time‐lapse images of droplet impact on smooth and different micropillar substrates at *P*_0_ = 50 bar and We = 6.5 to reveal different topology effects. a,e) smooth substrate and *p* = 80 µm, no bouncing with gas entrapment. b–d) *p* = 40–60 µm, bouncing with gas bubble entrapment in the droplet.

Using *P*_0_ = 50 bar as an example, **Figure** **3** (Video S2) shows time‐lapse images of droplet impact on the smooth and different superhydrophobic substrates. For the smooth substrate (Figure 3a) and micropillar substrate with *p* = 80 µm (Figure 3e), no droplet bouncing can be achieved at 50 bar. The droplet fluctuates after impacting and sticks on the substrate with some gas entrapment inside. Because of the relative low contact angle of the smooth substrate (*θ*_s_ ≈ 110°), the development of the capillary wave leads to asymmetrical movement of the top and bottom surface of the droplet, which results in the formation of a gas cavity inside the droplet during the recoil stage (i.e., 11 ms in Figure 3a). This captured gas cannot escape from the bottom, leading to the formation of an entrapped gas bubble on the substrate. However, from Figure 3b–e, it is noted that entrapped gas is not stationary and it moves together with the droplet moves. On micropillar substrates with *p* = 40–70 µm, however, droplet can bounce off the substrate. Better bouncing capability is observed on the substrate with *p* = 40–60 µm, i.e., no satellite droplet retention (Figure 3b–d), while there is satellite droplet remained on the substrates with *p* = 70 µm (Figure 1b).

**Figure 4:**
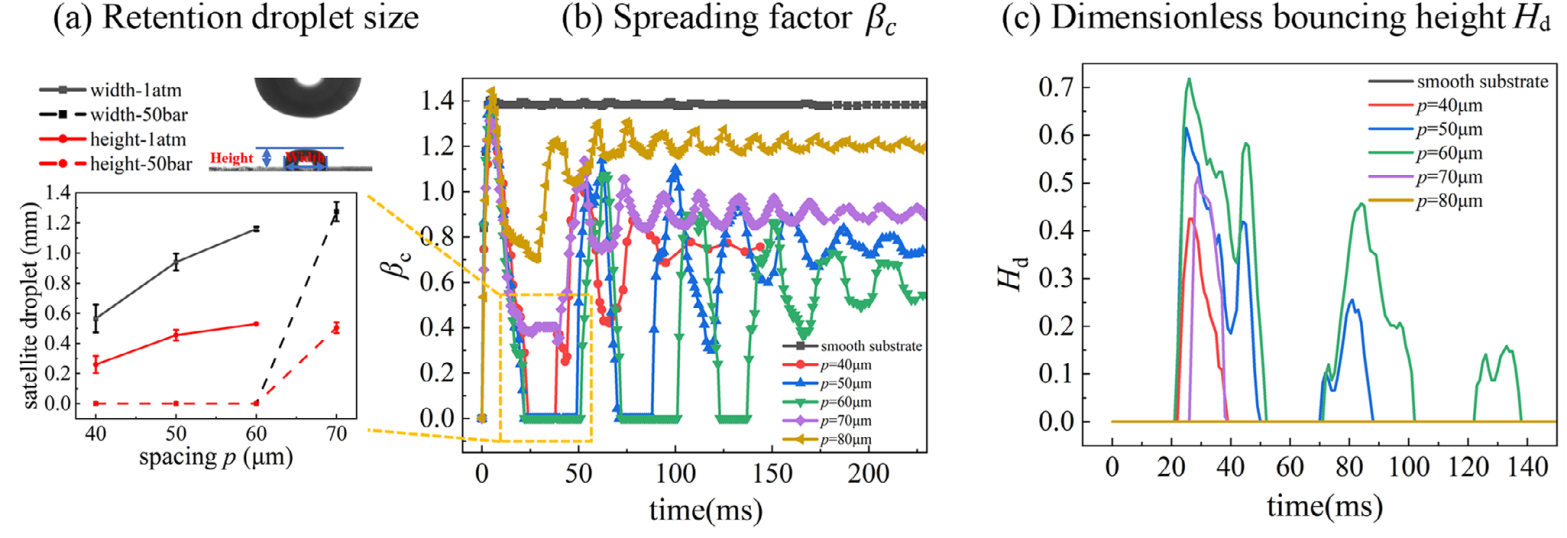
Quantitative descriptions (a) retention droplet size, b) spreading factor ***β***_***c ***_and c) dimensionless bouncing height *H*_d_) of 7droplet dynamics on smooth substrate and different micropillar substrates at *P*_0_ = 50 bar.

**Figure** **4** shows detailed droplet dynamics as well as the quantification of the retention droplets at 50 bar. The spacing between micropillars clearly affects significantly the droplet bouncing capability. At the same pressure, micropillar substrates with higher spacing distances result in larger satellite droplet retention, Figure 4a,b. It also shows that slightly smaller retention is obtained at higher ambient pressure. These retention droplets have irregular shaped, with an estimated volume ranging from 0.0254 to 0.38 µL, as characterized by both height and width in Figure 4a. Strangely droplet on substrate with *p* = 40–70 µm shows better bouncing characteristics, even though *p* = 80 µm has the best superhydrophobicity in terms of the measurement of the contact angle. With the same impacting outcome, i.e., bouncing with gas bubble entrapment but no satellite droplet retention (Figure 3b–d), droplet on substrate with *p* = 60 µm shows the best bouncing capability, i.e., larger bouncing height and more bouncing frequency compared with that on *p* = 40 and 50 µm at 50 bar, where are 1, 2 and 3 times bouncing on these three spacing substrates, respectively, Figure 4c. Clearly apparent contact angle cannot be used to directly describe the dynamic impact process. While it can reflect a qualitative or a general trend in static superhydrophobicity, quantitively it is the competition of different forces, relating to the capillary, dynamic pressure, and water hammer effects, which determines the possible transition from the Cassie to Wenzel state, as analyzed below. The morphology of the substrate also plays an important role, as to the capability of forming gas cavity, which also affect the overall bouncing capability.

### 2.3 Overall Result: The Phase Diagram

**Figure 5:**
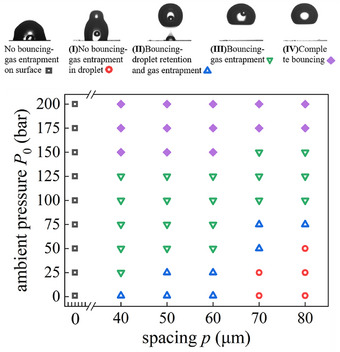
Phase diagram of droplet impact under different ambient pressures and spacing distances. (I) no bouncing with gas entrapment. (II) droplet bouncing with both satellite droplet retention and gas entrapment. (III) droplet bouncing with gas entrapment. (IV) complete droplet bouncing.

Three flow regimes were reported for droplet impact under 97 bar,^[^ ^20^ ^]^ i.e., complete droplet deposition, partial deposition and bouncing on smooth substrate. With improved visualization and high‐pressure environment chamber, we reveal the effects of ambient pressure on different impacting outcomes for all substrates, **Figure** **5**. For the smooth substrate, droplet cannot bounce and is always sticky on the substrate with an entrapped gas bubble inside, no matter how high the ambient pressure is. Four different regimes are identified on superhydrophobic substrates, i.e., no bouncing with gas entrapment (I), droplet bouncing with both satellite droplet retention and gas entrapment (II), droplet bouncing with gas entrapment (III), and complete droplet bouncing (IV). It shall be noted such a demarcation is based on pure experimental observation. To reach on this diagram, all experiments were performed a minimum of three times. There do have some variation in the border lines, where we pay extra attention and performed many more experiments. The border value, such as *P*_0_ = 50 bar for the transition of Regime II to III is based on statistical measures of 10 events on *p* = 50 µm substrate. Beyond the border lines, all the results are always consistent. For example, above 175 bar, one obtained always the same results, i.e., no satellite droplet retention and no gas bubble enclosure, regardless which superhydrophobic surface is used. The result clearly shows that the transition between different regimes is highly dependent on the ambient pressure and substrate topology. Micropillar substrate with higher spacing distances requires higher ambient pressures to reach better bouncing capability. For instance, for micropillar substrate with *p* = 40 µm, the transition to regime (III) is *P*_0_ = 25 bar, while for *p* = 50 and 60 µm, the required *P*_0_ is 50 bar. For these three spacing distances substrates, the regime (IV) is always achieved from *P*_0_ = 150 bar and above. For micropillar substrates with *p* = 70 and 80 µm, it needs relatively higher ambient pressure to realize the transition to higher flow regimes. With the increase of ambient pressure, the flow regime transit from (I) steadily into (IV) at *P*_0_ = 175 bar. Such dependence highlights the importance of surface topology in modulating droplet‐surface interactions.

### 2.4 Discussion

#### 2.4.1 The Phenomenological Model of Droplet Impact Under High Ambient Pressure

While there are many different phase diagrams have been identified for droplet impact under atmospheric pressures, the flow regime under high pressure is very rare. Under atmospheric conditions, both phenomena, i.e., satellite droplet retention and gas entrapment, have been identified under atmospheric conditions in different studies.^[^ ^23^, ^24^, ^25^, ^26^, ^27^, ^28^, ^29^, ^30^, ^31^, ^32^, ^33^ ^]^ In the work of Dupuy et. al.,^[^ ^20^ ^]^ three flow regimes for droplet impact at a maximum pressure of 97 bar, i.e., complete droplet deposition, partial deposition, and bouncing, under high pressures, based on some statistics analysis. However, due to the difficulties of visualization, no detailed impact phenomenon, such as droplet spreading dynamics, and gas entrapment phenomenon, could be identified.

We revealed in this work for the first time detailed droplet dynamics and identified four different flow regimes under high pressure up to 200 bar, from complete deposition to full bouncing. Clearly, the ambient pressure affects droplet dynamics significantly. It is believed that satellite droplet retention is the result of the transition from the Cassie state to the Wenzel state, leading to increased adhesion. On superhydrophobic substrates, the complex interplay among fluid dynamics, surface properties and liquid characteristics shall determine different outcomes. Other factors such as the presence of defects or non‐uniformity on the substrate, or environmental conditions such as humidity or local condensation could also reduce the repellency and promoting retention. For instance, the condensation induced droplet retention has been recently experientially proven on superhydrophobic substrates at low pressures.^[^ ^14^, ^18^ ^]^ These other factors, however, can be ruled out in this work, based on the fact that i) different substrates were produced by 3D printing, and all have regular shapes, as shown in Figure 8d, and ii) all experiments were performed under isothermal conditions with controlled moisture concentration under high pressures, so there is no evaporation or condensation as those occurring in low pressure. As to the gas bubble entrapment inside the droplet, it has been reported to be associated with two possible mechanisms. The first one is due to the gas film effect that can occur at the beginning of the impact.^[^ ^10^, ^12^, ^28^ ^]^ The droplet slides on a very thin air film sandwiched between the droplet and the substrate, and the liquid/solid contact causes the air film to be trapped, resulting in the formation of gas bubble inside the droplet. The second mechanism is related to the capillary wave occurring on the droplet surface, which may occur only within a specific range of Weber numbers. The capillary wave can cause the contact area center to dry out, and the recoil liquid then causes the closure of the air cavity, leading to gas bubble entrapment.^[^ ^22^, ^29^, ^30^, ^31^, ^32^, ^33^ ^]^ Further numerical simulations show that the second mechanism occurs in the Weber range of 5–10, and the air cavity can be formed either from the top or from the bottom part of the impact droplet, depending on the solid fraction of the superhydrophobic substrates.^[^ ^32^, ^33^ ^]^ Under high‐pressure conditions, both satellite droplet retention and gas entrapment are interactive and complex, occurring simultaneously in many conditions. The ambient pressure modifies significantly the interplay among fluid dynamics, surface properties, and liquid characteristics, leading to improved repellency. Several possible reasons can be accounted for high pressure, such as varied thermophysical properties, increased cushioning effect, increased viscous effect, and the interaction of droplet impact with different microstructures, which will be quantitatively analyzed in the next section. It is believed that the increased cushioning effect and hydrodynamic interaction of droplet with microstructures play a leading role in the formation of different flow regimes. The satellite droplet retention is decided by the force competition, and the Cassie state can be sustained if sufficient capillary pressure is maintained for a given ambient pressure. The possibility of gas entrapment depends mainly on the competition of forming gas cavities due to capillary wave, and the possible gas ventilation from the substrate. For smooth substrate, the lack of gas escaping route always and small capillary pressure always result in regime (I), i.e., droplet sticking to the surface with gas entrapment, Figure 5, which is very similar to that of Dupuy et. al.^[^ ^20^ ^]^

**Figure 6:**
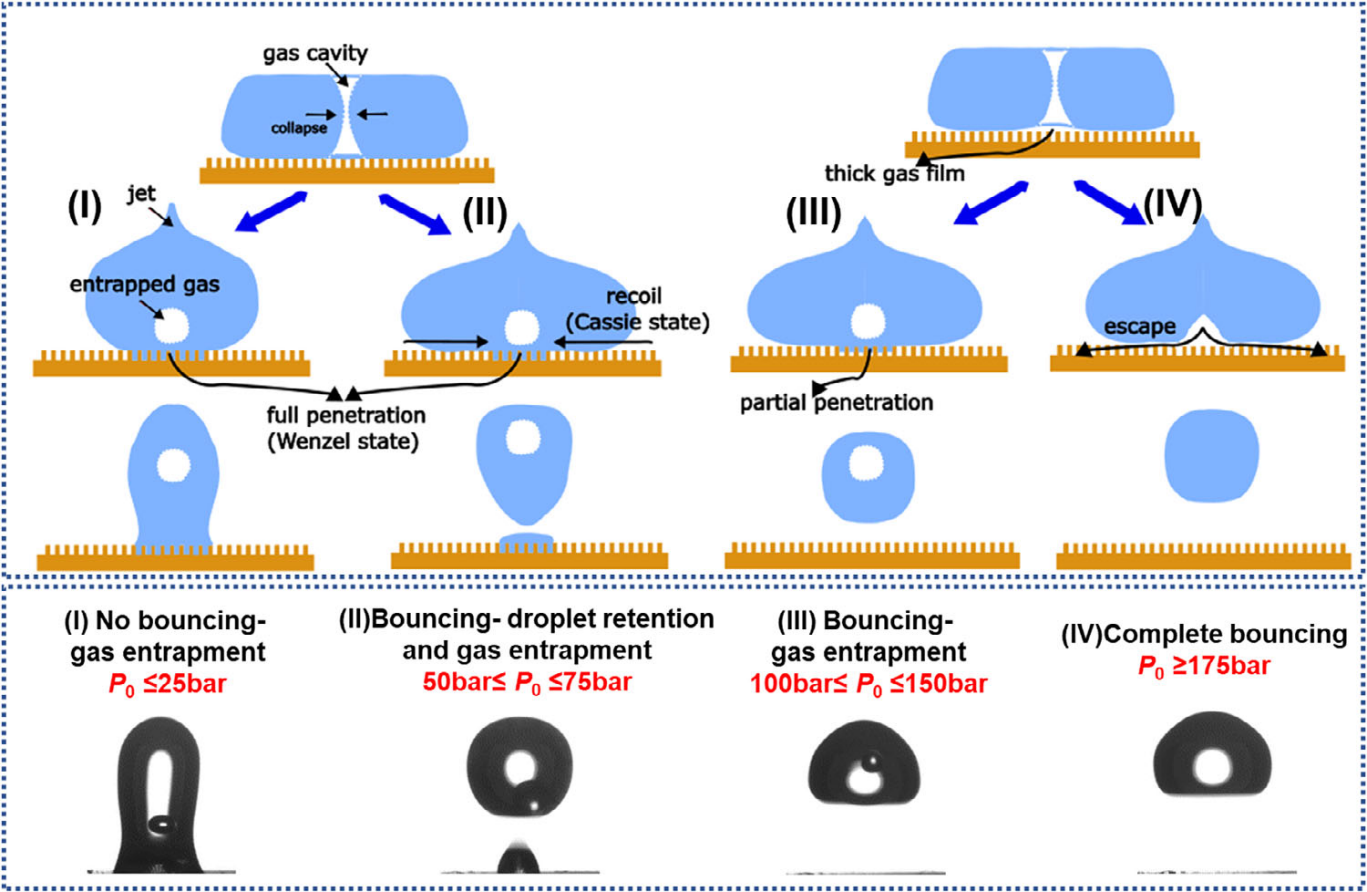
Mechanism of gas entrapment and satellite droplet retention on micropillar substrates under atmospheric pressure and high pressure (taking *p* = 70 µm as an example).

It appears that both the gas ventilation effect and capillary force increase significantly on superhydrophobic substrates due to the presence of different structures. Together with the increased cushioning effect at higher pressures, the droplet bouncing capability increases. Hence, a phenomenological model based on experimental observation is developed schematically in **Figure** **6**, using 70 µm substrate as an example, considering the effect of ambient pressure. From the observation, it is clear that the gas entrapment is due to the second mechanism, i.e., the effect of capillary wave and subsequent enclosure of a gas cavity. The observation show that a capillary wave develops upon droplet impact, and the top and bottom surface converge and form a gas cavity (7.6–8.3 ms shown in Figure 1a). As the droplet starts to recoil, the gas cavity is squeezed in the direction normal to the surface and merged with a gas film underneath. With further recoiling, the asymmetrical retraction between the top and bottom surface of the drop leads to the enclosure of the gas cavity, entrapping an air bubble inside the droplet, while simultaneously emitting a thin liquid jet (10 ms in Figure 1a). In most of the cases, the bottom surface of the droplet may penetrate into the substrate structures, forming liquid impalement, which prevents the leakage of the entrapped gas. It shall be noted that there are two possible scenarios of liquid impalement, i.e., fully or partially, influenced by the ambient pressure. Under *P*_0_ ≤ 25 bar, all the contacted surface can fully penetrate into micropillars, transiting into the Wenzel state, and consequently the droplet cannot bounce off (Regime I, Figure 6). As the pressure increases to *P*_0_ = [50, 75] bar, some contacted surface can still keep the Cassie state and continue to recoil. As a result, droplet can bounce off the substrate but leave a satellite droplet on the surface (Regime II, Figure 6). With further increase of the ambient pressure to 150 bar, the liquid impalement effect becomes weak, only partial impalement is observed due to increased cushioning effect associated with the increased pressure. The droplet can bounce off with no satellite droplet retention but still with gas bubble entrapment because of the disability to escape (Regime III, Figure 6). With the extreme high ambient pressure (≥175 bar), the gas cushioning effect becomes so strong that deforms bottom surface to create a dimple underneath the droplet. In the meantime, the high ambient pressure reduces the chances of forming gas cavities, which, even if formed, could be also vented out from the bottom surface through these micropillars during the recoil stage, leading to complete bouncing, i.e., no satellite droplet retention and no gas entrapment inside the droplet (Regime IV, Figure 6).

It shall be noted that under atmospheric pressure conditions, our observation is similar to the work of Nguyen and Ichiki^[^ ^22^ ^]^ and Xia et al.,^[^ ^32^ ^]^ which has also a similar We number range (We = 7–11). While two types of gas bubble entrapment phenomena were observed under 1 atm conditions,^[^ ^30^ ^]^ i.e., bubble stay at the bottom of the droplet or float inside the droplet for high superhydrophobic case, i.e., with apparent contact angle of 161°. Under high pressure, only the floating bubble model is observed, possibly due to the combined ambient pressure and high superhydrophilicity characteristics. Different to the scenario under atmospheric pressure, Figure 6 extends the scenario into 200 bar regime.

#### 2.4.2 The Effect of Gas Cushion and Hydrodynamic Impact

A general trend observed in this work is the disappearance of satellite droplet retention and gas entrapment as the ambient pressure reaches to a critical value, which is highly substrate topology dependent. It shall be noted that transition pressure between different flow regimes changes slightly for different substrates, as shown in Figure 5. For instance, the reduction of the spacing from 70 to 60 µm, the critical pressure to entering regime (IV) is dropped from 175 to 150 bar. It is expected that a decrease in spacing increases the capillary effect, hence leading to complete bouncing at lower ambient pressure. It is also noted that considering all substrates, the best one in achieving complete bouncing, is *p* = 60 µm (with lower critical pressure and more bouncing frequency). Surprisingly, it does not have the highest stationary contact angle. It shall be cautious to apply the value of contact angle as a universal index for wettability. Clearly droplet bouncing capability is heavily affected by the hydrodynamic impacting process, where ambient pressure plays a leading role, that calls for a careful design of the substrate topology for different applications.

It is clearly that the increase of ambient pressure shall indirectly affect satellite droplet retention and gas entrapment by modulating the air layer thickness, stability, and the gas‐liquid interactions. Assuming a smooth substrate, the initial air film thickness *H* can be calculated as.^[^ ^34^, ^35^ ^]^

(1) $$H=R_{0}\;St^{2/3}\varepsilon^{\left(2-\gamma \right)/\left(2\gamma -1\right)}$$

where *R*_0_, St, ɛ, γ are initial droplet radius, Stokes number, gas compressibility factor, and specific heat ratio, respectively. The equation is obtained by approximating the impact as a transient and adiabatic process, where γ is 1.4 for air or N_2_ environment. St and ɛ can be calculated as

(2) $$\mathrm{St}=\mu_{g}\;/\left(\rho_{l}R_{0}V_{0}\right)$$

(3) $$\varepsilon \equiv P_{0}/{\left(R_{0}{\mu_{g}}^{-1}V_{0}^{7}\rho_{l}^{4}\right)}^{1/3}$$

Taking physical properties (µ_*g*_, dynamic viscosity of gas and ρ_*l*_ density of droplet), impact velocity *V*_0_ and ambient pressure *P*_0_ (1 atm, 0.4071 m ^−1^s and 200 bar, 0.3680 m ^−1^s) into Equation (1), the air film thickness *H* can be obtained, which is ≈3.81 µm at 1 atm and ∼25.86 µm at 200 bar. It is clear that as the ambient pressure increases, the average air film thickness increases saliently, i.e., ≈7 times thicker film is expected at 200 bar. This dense and thicker gas film increases greatly the air cushioning effect, which would prevent the penetration of the droplet into the substrate, hence minimizing the chance of satellite droplet retention. However, this enhanced cushioning effect is not the only reason, as the experiment shows clearly that even at the ambient pressure of 200 bar, no droplet bouncing can be achieved for the smooth substrate. Considering the high pressure and adiabatic impact on regular micropillars in this work, other possibilities such as re‐condensation and substrate default can be ruled out. The interaction of hydrodynamic impact with the substrate topology appears to be responsible for the disappearance of the satellite droplet.

It is clear that the transition into the Wenzel status is highly dependent on the competition among different forces, notably the impact force, the water hammer pressure^[^ ^23^, ^24^, ^36^, ^37^, ^38^ ^]^ and the capillary pressure^[^ ^39^, ^40^ ^]^ arising from the substrate.

The dynamic pressure (*P*_d_) can be calculated as

(4) $$P_{d}=\frac{1}{2}\rho V_{0}^{2}$$

The capillary pressure (*P*_c_), which is essential to prevent the droplet to penetrate into the substrate, resulting in so called impalement, can be calculated as.^[^ ^39^ ^]^

(5) $$P_{c}=-\sigma \cos\theta_{A}L_{c}/A_{c}$$

where *L_c_* and *A_c_* are the capillary perimeter and area, respectively. θ_*A*_ is the Young's advancing contact angle on smooth substrate (here θ_*A*_ ≈120° as measured). For a substrate with micropillar structures shown in Figure 8c, *P*_c_ can be calculated as

(6) $$P_{c}=-\sigma \cos\theta_{A}\left(4f/a\left(1-f\right)\right)$$

The calculated *P*_c_ is ≈2333, 1333, 875, 622, and 467 Pa for micropillar substrates with spacing *p* = 40, 50, 60, 70 and 80 µm, respectively.

The water hammer pressure (*P*_WH_) is produced by the sudden stop of droplet by the substrate, which is related with water compressibility ability and can be calculated as.^[^ ^38^ ^]^

(7) $$P_{\mathrm{WH}}=k\;\rho {\mathrm{CV}}_{0}$$

where *C* is the speed of sound in liquid (≈1482 m s^−1^ in water). Considering the well‐established capillary and impact forces, the prediction of water hammer effect, which relates to the liquid compressibility, remains as the largest uncertainty in the force analysis. This uncertainty has been considered by using different values of the water hammer coefficient, *k*. A constant value of 0.2 is usually used in some literature^[^ ^36^, ^38^ ^]^ for droplet impacting on smooth substrate. However, it has been suggested that *k* = 0.2 overrated water hammer pressure effects on droplet impact, especially on superhydrophobic substrates. Different coefficients have been suggested such as *k* ≈0.001^[^ ^41^, ^42^ ^]^ and ≈0.003^[^ ^37^, ^43^ ^]^ for any substrate. Considering that the water hammer effect shall be related to substrate topology, a constant value would not be suitable for all substrates. It would be expected that there shall have some dependence of the *k* value to the substrate geometry. One of such effort was conducted by Dash et al.^[^ ^23^ ^]^ who considered the dependence of *k* on *P*_c_ based on force balance analysis, i.e., considering the force balance among the wetting pressure *P*_d_ and *P*_WH_ and the anti‐wetting pressure *P*_c_ together. One correlation of *k*‐ *P*_c_ was proposed, *k* = 2.54 × 10^−7^ *P*_c_ + 7.53 × 10^−4^, and ≈2 orders of magnitude lower *k* was found on a micro‐nanostructured substrate compared to that of a smooth substrate. With such a modification, the force analysis became consistent with their experimental observation. However, it shall be noted that such a conclusion was found under atmospheric pressure conditions. Also due to their special design of hybrid surface, the validity of the correlation needs to be re‐examined on micropillar substrates under high pressures.

**Figure 7:**
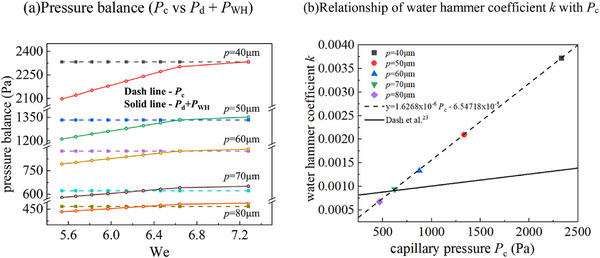
A new correction of water hammer coefficient *k* with capillary pressure *P*_c_. a) Pressure balance of *P*_c_ versus *P*_d_ + *P*_WH_. b) The linear relationship of water hammer coefficient *k* with capillary pressure *P*_c_.

However, using the same *k‐ P*_c_ correlation^[^ ^23^ ^]^ developed under atmospheric pressure conditions, all the calculations showed a much larger capillary force comparing with the sum of impacting and hammer pressure, which clearly contradicts to our experimental observation of satellite droplet retention. To prevent the transition from the Cassie state to the Wenzel state, the anti‐wetting pressure (*P_c_*) shall be larger than the sum of wetting pressure (*P*_d_ + *P*_WH_), i.e., *P*_c_ >*P*_d_ + *P*_WH_ and a critical Weber number, We_c_, is defined as *P*_c_ = *P*_d_ + *P*_WH_. Using the experimental results in Figure 5 as the input, a new correlation, *k* = 1.6268 × 10^−6^ *P*_c_ – 6.54718 × 10^−5^ where *P*_c_ has the unit of Pa, is developed that suitable for high pressure applications, as shown in **Figure** **7**.

The developed new correlation shows a linear dependence of *k* on *P*_c_, which is similar to the previous work,^[^ ^23^ ^]^ but have different coefficients. With such modified water hammer coefficients, we could explain quantitatively the effect of different substrates, and different ambient pressures required for the transition into Regime (III). It also qualitatively explains why the increase of spacing has a general trend of increasing the retention droplet size for a given pressure, as shown in Figure 4a.

## 3 Conclusion

This study presents the first experimental investigation of droplet impact under high ambient pressure (up to 200 bar) onto different superhydrophobic substrates in the low Weber number regime (We = 5–10). A phenomenological mode is developed taking into the consideration of both enhanced cushioning effect and hydrodynamic impact dynamics at high pressure. The major conclusions can be summarized as:

- Four different regimes are identified for droplet impact under high pressures, i.e., no bouncing with gas entrapment (I), droplet bouncing with both satellite droplet retention and gas entrapment (II), droplet bouncing with gas entrapment (III), and complete droplet bouncing (IV).
- A general trend is that droplet bouncing capability increases with the increase of ambient pressure, and complete bouncing is achieved for all substrates at *P*_0_ ≥ 175 bar. However, the transition into different regimes is affected by both ambient pressure and substrate topology.
- For a given structured superhydrophobic substrate, the flow regimes transits from (I) to (IV) steadily with the increase of ambient pressure, while for the smooth substrate, only the flow regime – no bouncing, is achieved regardless of the pressure. While for a given pressure, increasing space among micropillars (i.e., decrease of the solid fraction) leads to large satellite droplet retention.
- With a modified water hammer coefficient, the hydrodynamic impact model can be used to explain the disappearance of the satellite droplet, i.e., the increased capillary effect from the substrate, as evidenced by the spacing between micropillars.

Such work advances our droplet impact study into 200 bar domain, which is of high relevance to a number of high‐pressure applications such as deep‐sea separation and high‐pressure atomization. However, it shall be noted that the current study is limited to water droplets and a specific range of surface topologies under a fixed impact height. Future work could explore the effect of varying Weber numbers and liquids, with more complex surface designs to further elucidate the interplay between ambient pressure and surface topology.

## 4 Experimental Section

### Experimental System

**Figure 8:**
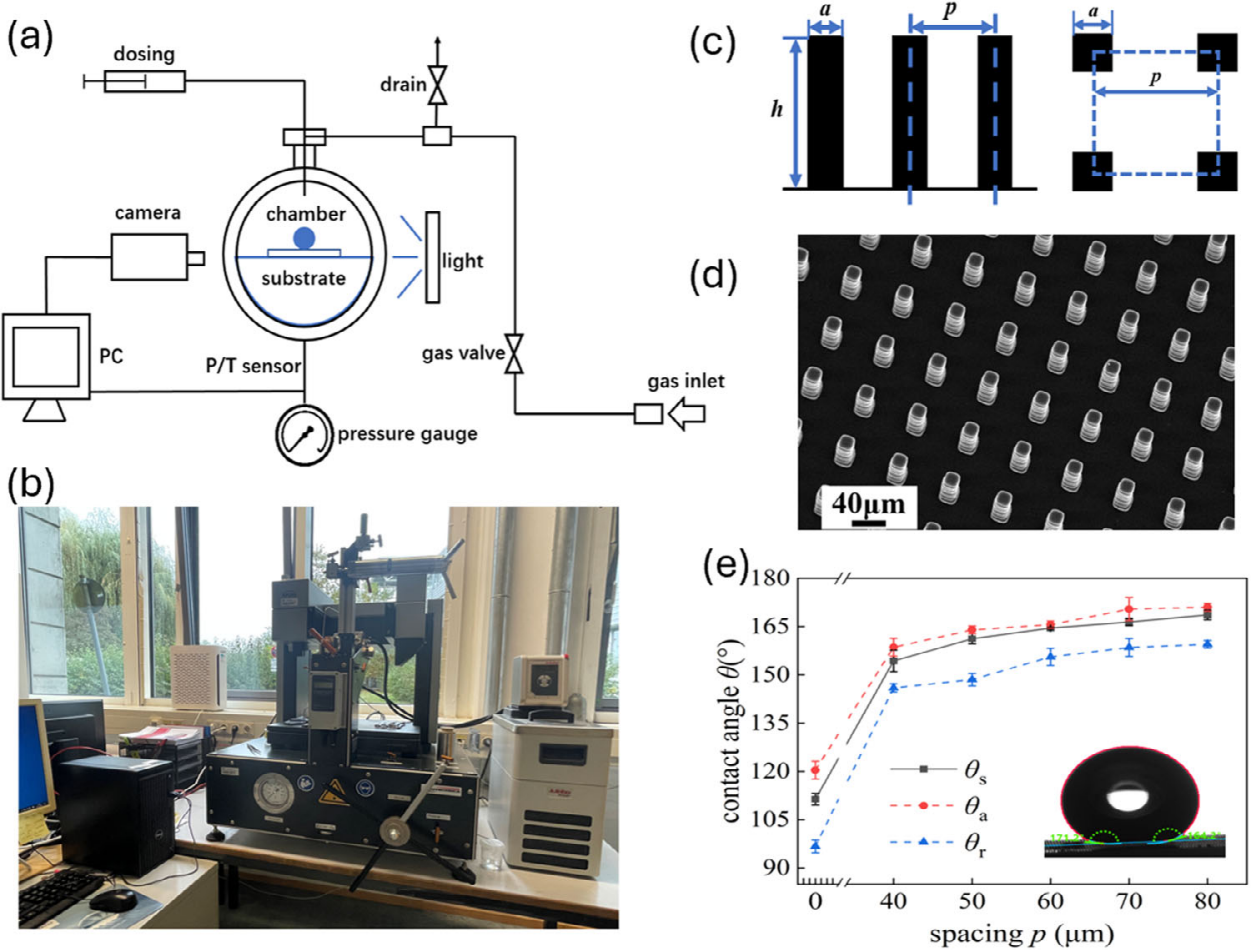
Experimental method: a) Schematic diagram of the high‐pressure droplet impact experimental setup, b) Experimental bench, c) Geometrical parameters of micropillar substrates. d) SEM images of tilt view of micropillar substrate with [*a*, *p*, *h*] µm = [20,50,80] µm and e) Contact angles of DI water on different substrates.

To examine clearly the droplet impact and impact under high pressure, we have used a completely different system comparing to Dupuy et. al.,^[^ ^20^ ^]^ which is equipped with a high‐pressure environmental chamber, a large transparent observation window and an integrated high‐speed CCD camera at 1000–3000 frames per second. Specifically, the droplet impact experiments were conducted with a High‐Pressure Pendant Drop (PD) Apparatus (PDE‐1400MD, Eurotechnica GmbH, Germany), shown in **Figure** **8**a,b. The system includes a high‐pressure environment chamber up to 250 bar, a droplet delivery system, and various regulation valves and sensors. Nitrogen gas, delivered from high‐pressure gas cylinders, was used to pressurize the chamber, where the pressure was precisely controlled by a pressure sensor. In the experiments, the ambient pressure *P*_0_ was controlled in the range of [1 atm – 200 bar] and temperature was controlled under T = 21 ± 1.5 °C. Deionized water was used as the impacting fluid. Droplets was delivered from a fixed height by the dosing system and controlled at an initial diameter *D*_0_ of 3.06 ± 0.04 mm. The impact velocity *V*_0_ was calculated as 0.3680‐0.4071 m ^−1^s, corresponding to Weber number (We) of 5.5–7.1, where We is defined as We = $\rho V_{0}^{2}D_{0}/\sigma$, with ρ and σ the droplet density and surface tension, respectively. Please refer to Supporting information 3 on detailed values of impacting velocity and We number by considering the large variations of gas density and other properties in different pressures. The chamber was equipped with optical windows and the droplet dynamics was obtained from the side view using a high‐speed camera at 1000–3000 frames per second.

### Substrate Fabrication

Considering the importance of substrate in regulating droplet impact dynamics, both smooth and structured superhydrophobic substrates were prepared. Micropillar substrates were fabricated by the projection micro‐stereolithography 3D printing technique (S230, BMF, USA). The printing material was photosensitive resin. As shown in Figure 8c, the pillar length *a* and height *h* were fixed at 20 and 80 µm, respectively. The spacing distance *p* was set at 0 (smooth), 40, 50, 60,70 and 80 µm. The solid fraction *f* on a textured substrate which is an important parameter in regulating droplet dynamics, could be calculated as *f* = *a* ^2^ /*p* ^2^. To engineer different wettability, all substrates were coated by a 2% solution of 1H,1H,2H,2H‐perfluorodecyltriethoxysilane in hexane (Sigma‐Aldrich) for one day and then heated at 60 °C for 2 h.

Scanning electron microscopy (EM‐30N, COXEM, Korea) was used to characterize the topography of the substrates (shown in Figure 8d). A layer of Au was sputtered on the substrates with a current 7 mA to facilitate visualization. The acceleration voltage of 15 kV and spot size of 7 were used with electron detectors.

The wettability of water droplets was measured by a Drop Shape Analyzer (DSA100, Krüss, Germany). Static contact angle *θ_s_* was measured by dispensing a sessile drop on the fabricated substrates. Dynamic contact angles (i.e., advancing contact angle *θ*_a_ and receding contact angle *θ*_r_) were measured by the tilting base method. Both advancing and receding contact angles were recorded when the droplet was going to roll down. All the measurements were conducted three times. The contact angles of these micropillar substrates are shown in Figure 8e. It shows that contact angle increases with the raised spacing distance, and all micropillar substrates achieve the superhydrophobic characteristics.

## Conflict of Interest

The authors declare no conflict of interest.

## Supporting information



Supporting Information

Supplemental Video 1

Supplemental Video 2

## Data Availability

The data that support the findings of this study are available in the supplementary material of this article.
