# Supplementary material for: Droplet Impact on Superhydrophobic Surfaces Under High Pressures
Source: Small Methods. 2025 Jul 25;10(3):2500913. doi: 10.1002/smtd.202500913 (PMC12893251; doi:10.1002/smtd.202500913)
Supplement: Supplementary file 1 — Supporting Information [file SMTD-10-2500913-s003.docx]

## **Supporting Information**

**Supporting information 1**: Videos show droplet impact on a substrate with [*a*, *p*, *h*] = [20, 70, 80] $\mu m$ with a solid fraction of *f* = 0.082 under a range of ambient pressures, *P*_0_ = 1atm, 50bar, 100bar and 175bar. The static, advancing and receding contact angles are $\theta$_s_= 166.4$\pm$1.17°, $\theta$_a_= 170.4$\pm$3.61° and $\theta$_r_= 158.5±2.81°, respectively.

**Supporting information 2:** Videos of of the droplet impact on smooth and different micropillar substrates at *P*_0_ = 50 bar to reveal different topology effects.

## **Supporting information 3: Calculation of impact velocity and Weber number**

According to Newton's second law, the relationship between velocity and air resistance can be calculated as

$m\frac{dv}{dt}=mg-F_{d}$ (1)

where m, v and g are mass, velocity and gravity of droplet, respectively. F_d_ is air resistance, calculated as

$F_{d}= \frac{1}{2}C_{d}\rho_{g}Av^{2}$ (2)

where A is windward area, approximately $\pi r^{2}$. $\rho_{g}$ is density of ambient gas, at high pressure which is proportional to that at atmospheric pressure.

C_d_ is air resistance coefficient, up to Reynolds number (Re)

$Re=\rho_{air}v D_{0}/\mu_{air}$ (3)

When at transition regime ($1<Re$<1000), C_d_ can be calculated as

$C_{d}=\frac{24}{Re}(1+0.15Re^{0.687})$ (4)

Otherwise at laminar flow (Re>1000), C_d_ ≈0.47.

Impact velocity and We at different pressures shows in Figure S1., with the error are 10% and 20% at 1atm and 200bar, respectively.


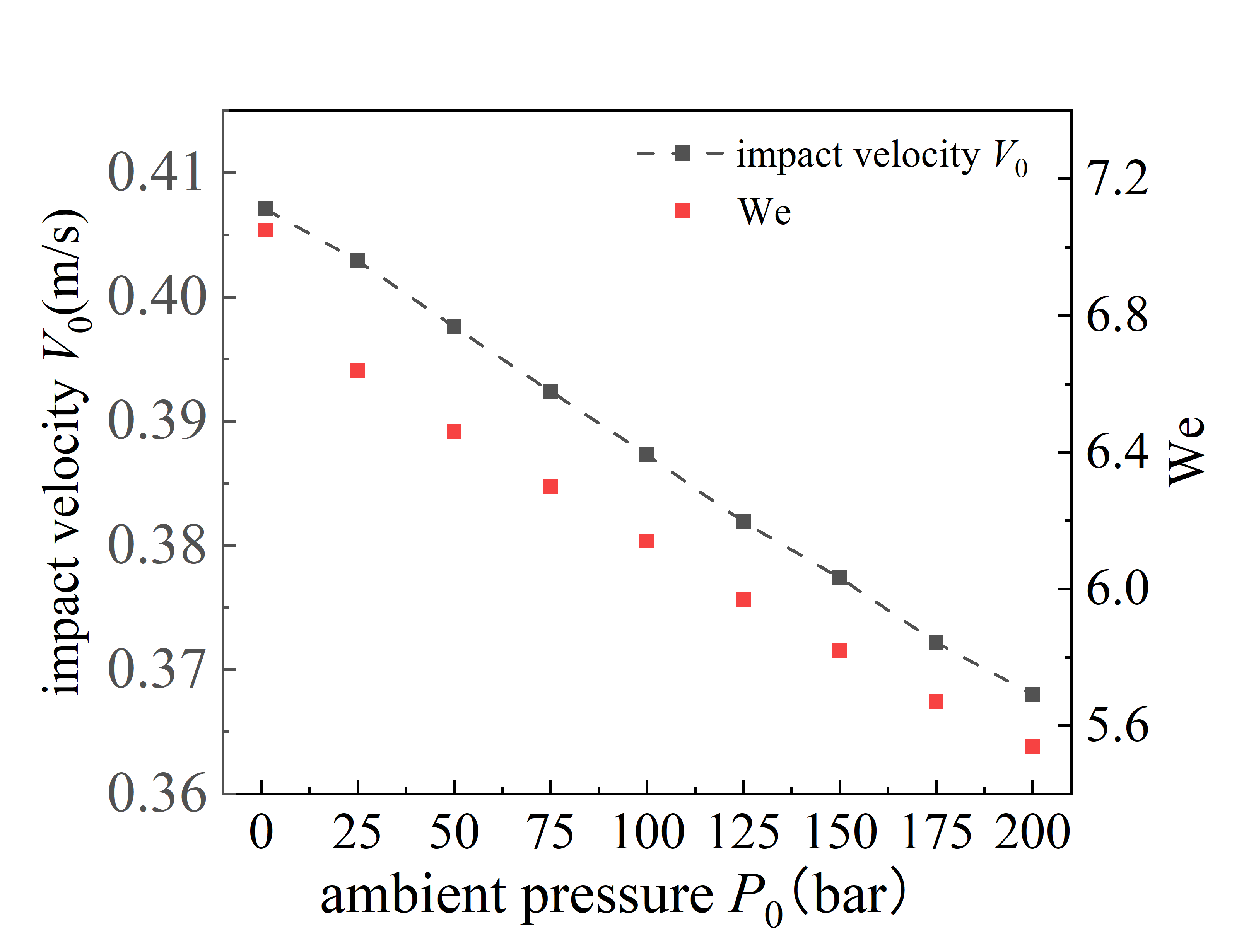


Figure S1. Impact velocity and We at different pressures.
